# The presence, nature and network characteristics of behavioural phenotypes in temporal lobe epilepsy

**DOI:** 10.1093/braincomms/fcad095

**Published:** 2023-03-30

**Authors:** Aaron F Struck, Camille Garcia-Ramos, Veena A Nair, Vivek Prabhakaran, Kevin Dabbs, Melanie Boly, Lisa L Conant, Jeffrey R Binder, Mary E Meyerand, Bruce P Hermann

**Affiliations:** Department of Neurology, University of Wisconsin-Madison, Madison, WI 53726, USA; Department of Neurology, William S. Middleton Veterans Administration Hospital, Madison, WI 53705, USA; Department of Neurology, University of Wisconsin-Madison, Madison, WI 53726, USA; Department of Radiology, University of Wisconsin-Madison, Madison, WI 53726, USA; Department of Neurology, Medical College of Wisconsin, Milwaukee, WI 53226, USA; Department of Neurology, University of Wisconsin-Madison, Madison, WI 53726, USA; Department of Neurology, University of Wisconsin-Madison, Madison, WI 53726, USA; Department of Neurology, Medical College of Wisconsin, Milwaukee, WI 53226, USA; Department of Neurology, Medical College of Wisconsin, Milwaukee, WI 53226, USA; Department of Medical Physics, University of Wisconsin-Madison, Madison, WI 53726, USA; Department of Neurology, University of Wisconsin-Madison, Madison, WI 53726, USA

**Keywords:** temporal lobe epilepsy, phenotypes, behaviour, psychopathology

## Abstract

The relationship between temporal lobe epilepsy and psychopathology has had a long and contentious history with diverse views regarding the presence, nature and severity of emotional–behavioural problems in this patient population. To address these controversies, we take a new person-centred approach through the application of unsupervised machine learning techniques to identify underlying latent groups or behavioural phenotypes. Addressed are the distinct psychopathological profiles, their linked frequency, patterns and severity and the disruptions in morphological and network properties that underlie the identified latent groups. A total of 114 patients and 83 controls from the Epilepsy Connectome Project were administered the Achenbach System of Empirically Based Assessment inventory from which six Diagnostic and Statistical Manual of Mental Disorders-oriented scales were analysed by unsupervised machine learning analytics to identify latent patient groups. Identified clusters were contrasted to controls as well as to each other in order to characterize their association with sociodemographic, clinical epilepsy and morphological and functional imaging network features. The concurrent validity of the behavioural phenotypes was examined through other measures of behaviour and quality of life. Patients overall exhibited significantly higher (abnormal) scores compared with controls. However, cluster analysis identified three latent groups: (i) unaffected, with no scale elevations compared with controls (Cluster 1, 37%); (ii) mild symptomatology characterized by significant elevations across several Diagnostic and Statistical Manual of Mental Disorders-oriented scales compared with controls (Cluster 2, 42%); and (iii) severe symptomatology with significant elevations across all scales compared with controls and the other temporal lobe epilepsy behaviour phenotype groups (Cluster 3, 21%). Concurrent validity of the behavioural phenotype grouping was demonstrated through identical stepwise links to abnormalities on independent measures including the National Institutes of Health Toolbox Emotion Battery and quality of life metrics. There were significant associations between cluster membership and sociodemographic (handedness and education), cognition (processing speed), clinical epilepsy (presence and lifetime number of tonic–clonic seizures) and neuroimaging characteristics (cortical volume and thickness and global graph theory metrics of morphology and resting-state functional MRI). Increasingly dispersed volumetric abnormalities and widespread disruptions in underlying network properties were associated with the most abnormal behavioural phenotype. Psychopathology in these patients is characterized by a series of discrete latent groups that harbour accompanying sociodemographic, clinical and neuroimaging correlates. The underlying neurobiological patterns suggest that the degree of psychopathology is linked to increasingly dispersed abnormal brain networks. Similar to cognition, machine learning approaches support a novel developing taxonomy of the comorbidities of epilepsy.

## Teaser

Struck *et al*. demonstrate that while temporal lobe epilepsy patients (*n* = 114) exhibit significantly abnormal scores across measures of psychopathology compared with controls (*n* = 83), cluster analysis identifies three latent patient groups characterized by varying levels of emotional–behavioural distress ranging from none to severe, the clusters linked to sociodemographic, clinical, imaging and network characteristics.

## Graphical Abstract

**Graphical Abstract:**
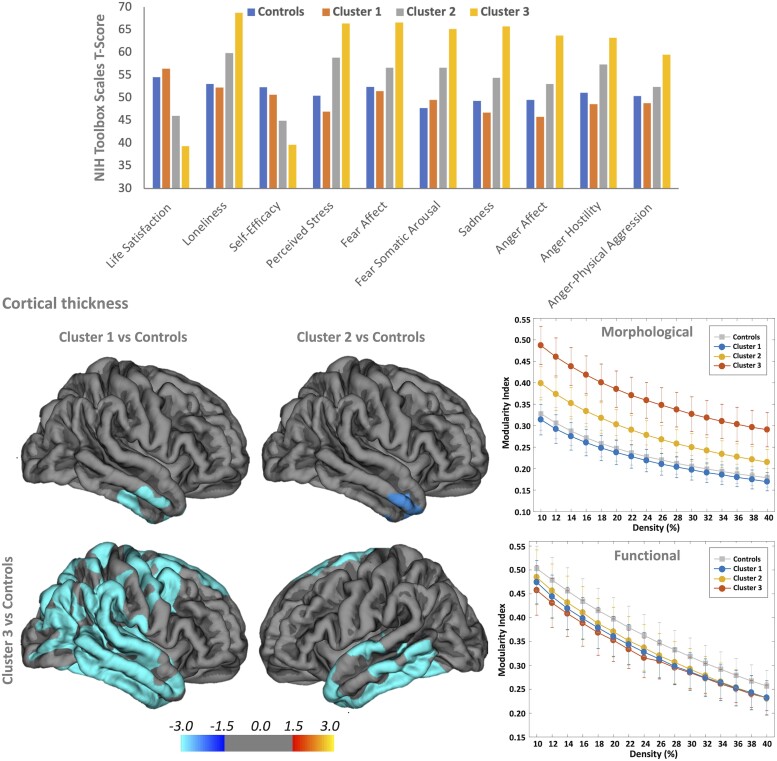

## Introduction

A substantial population-based and clinical literature has documented the increased risk of cognitive, behavioural and social complications of the epilepsies including their rates of occurrence and the clinical, sociodemographic and neuroimaging correlates that accompany them.^1-3^ These neurobehavioural risks and their associated burdens, including on life course, are widely recognized and have been addressed by national and international reports, commissions and task forces.^4,5^

Among the neurobehavioural comorbidities, an especially controversial issue has been the relationship between psychopathology and temporal lobe epilepsy (TLE). This relationship, initially proposed in the mid-20th century,^6^ was provocative and suggested that TLE could provide a unique neurobiological window through which to understand abnormal behaviour. The debate over the ensuing decades coursed through specific behaviours (e.g. aggression),^7^ Diagnostic and Statistical Manual of Mental Disorders (DSM)- or International Classification of Diseases (ICD)-based psychiatric diagnoses (e.g. depression and schizophrenia),^8^ non-psychopathological personality and behavioural changes (e.g. hypergraphia and hyperreligiosity)^9-11^ and a diversity of patient and/or proxy-completed symptom inventories (e.g. Minnesota Multiphasic Personality Inventory, General Health Questionnaire and Beck Depression Inventory).^12-14^ Importantly, the fundamental question and debate centred on whether patients with TLE, as a group, were at increased risk compared with other epilepsy syndromes and/or controls as well as whether there were specific clinical features (e.g. age of onset, duration and laterality) that might underlie any observed heterogeneity in behavioural presentations.

Driven in large part by the presence of significant heterogeneity in the cognitive presentations of patients with TLE, application of unsupervised machine learning approaches has begun to identify underlying groups of patients or latent cognitive phenotypes that capture neuropsychological variability in a patient-centred fashion. Efforts to date have largely focused on cognition in adults with TLE, and collectively these studies indicate that TLE is characterized by three to four phenotypes including one with an unexpected intact cognitive profile, one with an unanticipated (for focal epilepsy) profile of generalized cognitive abnormality and one to two groups associated with more expected (for TLE) cognitive anomalies (e.g. memory and/or language/executive dysfunction). The linked structural, diffusion, resting state and mesoscale anomalies associated with these cognitive phenotypes have been characterized.^2^

Much more slowly developing are comparable approaches to the behavioural complications of the epilepsies. One investigation examined adults with TLE,^15^ one focused on children with TLE,^16^ and the third examined children with new onset focal and generalized epilepsies.^17^ Three clusters have been identified across these studies including one group behaviourally comparable with controls, one highly distressed group with pathology across all behavioural measures and an intermediate group with elevated but less striking patterns of distress. The imaging approaches to date have been limited to structural assessment and have been largely unrevealing. Yet to be explored are the potential network disruptions that may underlie the identified behavioural groups including the use of multimodal imaging techniques and the application of graph theory (GT) analytics to elucidate the nature of those disrupted networks.

Here, we address these issues in the Epilepsy Connectome Project (ECP)—a well-characterized cohort of patients with TLE and controls assessed through multiple imaging modalities, a wide range of clinical and sociodemographic variables and diverse behavioural and cognitive measures. The aims of this investigation were to (i) determine whether latent behavioural groups could be identified in patients with TLE, (ii) characterize their demographic, clinical epilepsy and cognitive correlates and (iii) identify the network disruptions that may underlie the identified behavioural phenotypes.

### Participants

Participants included 114 patients with TLE and 83 healthy controls. Data collection occurred from March 2016 to December 2018 at the Medical College of Wisconsin and the University of Wisconsin-Madison. All participants provided written informed consent for the study. The project was approved by the respective institutional review boards and was performed in accordance with the Declaration of Helsinki.

Eligible TLE participants were between the ages of 18 and 60, had tested full-scale intelligence quotient (FSIQ) at or above 70, spoke English fluently and were without medical contraindications to MRI. Potential cases were reviewed by the study epileptologists, which included all available current and past medical records including clinical seizure descriptions, interictal and ictal EEGs, neuroimaging and treatment. If there was any uncertainty regarding whether a potential participant met inclusion criteria, they were discussed by the epileptologists in a research meeting to reach consensus. The diagnosis of TLE was supported by two or more of the following: (i) described or observed clinical semiology consistent with seizures of temporal lobe origin, (ii) EEG evidence of either Temporal Intermittent Rhythmic Delta Activity or temporal lobe epileptiform discharges, (iii) temporal lobe onset of seizures captured on video EEG monitoring or (iv) MRI evidence of mesial temporal sclerosis or hippocampal atrophy. Patients with any of the following were excluded: (i) lesions other than mesial temporal sclerosis causative for seizures and (ii) an active infectious/autoimmune/inflammatory aetiology of seizures. A goal of the ECP is to understand the phenotypical heterogeneity in the general population of TLE, not just the intractable and surgical cases and uses the common criteria that an epileptologist would use in clinic to decide if a patient has TLE, namely structural brain MRI, interictal EEG and clinical semiology and ictal-EEG monitoring in the 34% of patients who needed epilepsy monitoring unit (EMU) admission either to clarify diagnosis or in anticipation of epilepsy surgery. An intent of the ECP is to compare morphological, diffusion-weighted imaging (DWI) and functional connectivity in TLE and controls making it desirable to exclude patients with overt structural lesions that would hinder this analytical approach. Overall, from 16% to 45% had hippocampal atrophy depending on the adjusted [age, gender and intracranial volume (ICV)] *Z*-score threshold (−1.5 or −1.0) pointing to the presence but less severe nature of hippocampal atrophy in this TLE group; the others considered non-lesional TLE.

Control participants were healthy adults between the ages of 18 and 60. Exclusion criteria included (i) Edinburgh Laterality (handedness) Quotient less than +50; (ii) primary language other than English; (iii) history of any learning disability, brain injury or illness; (iv) substance abuse or major psychiatric illness (major depression, bipolar disorder or schizophrenia); (v) current use of vasoactive medications; and (vi) medical contraindications to MRI.

## Materials and methods

### Behavioural measures

All participants completed the Achenbach System of Empirically Based Assessment (ASEBA), a 126-item self-report questionnaire for adults (ages 18–59) that provides standard scores for the following DSM-oriented scales: depressive problems, anxiety problems, somatic problems, avoidant personality problems, attention deficit/hyperactivity problems (inattention and hyperactivity/impulsivity subscales) and antisocial personality problems. Items are scored on a 3-point rating scale: 0 (not true), 1 (somewhat or sometimes true) and 2 (very true or often true). Higher raw scores indicate more problematic behaviour and normalized age- and sex-adjusted *T*-score are derived. In addition to providing continuous measures for analysis, clinical interpretations of scale elevations are available (e.g. *T*-scores ≥ 70 are abnormal; *T*-scores 65–69 are borderline). The ASEBA is a reliable and valid measure for the 18–59 general population with specific information on the DSM oriented scales available online (https://aseba.org/research/aseba-dsm-5-oriented-scales/).

### Cluster analysis

Standardized adjusted *T*-scores for the six DSM syndrome scales were used for hierarchical clustering among patients with TLE. The optimal clustering method was determined as in our previous investigation.^15^ In short, we maximized the agglomerative coefficient by comparing different linkages (‘cluster’ 2.1.0 R package ‘Finding Groups in Data’: Cluster Analysis Extended).^18^ This was followed by determining the optimal number of clusters using the gap statistic, which was maximized using the method proposed by Tibshirani *et al*.^19^ such that the optimal cluster number is the lowest cluster number within 1 standard error of the local maximum. Then, hierarchical cluster bootstrapping with replacement for 1000 trials was used to ensure stability of clustering, and final partitions were determined by the frequency of concurrence over the 1000 trials (‘fpc’—Flexible Procedures for Clustering 2.2–3, Christian Henning R package). All statistical analysis was performed in R version 4.0.2

### Image acquisition

MRI was performed on 3T GE 750 scanners at both institutions. Details regarding imaging acquisition can be found in our previous publication.^20^ In summary, the parameters for *T*_1_-weighted images were as follows: repetition time (TR)/echo time (TE) = 604 ms/2.516 ms, inversion time (TI) = 1060.0 ms, flip angle = 8°, field-of-view (FOV) = 25.6 cm, 0.8 mm isotropic; parameters of Cube *T*_2_-weighted images were as follows: TR/TE = 2500 ms/94.641 ms, flip angle = 90°, FOV = 25.6 cm, 0.8 mm isotropic; and parameters of resting-state functional MRI (rs-fMRI) were as follows: 8 bands, 72 slices, TR/TE = 802 ms/33.5 ms, flip angle = 50°, matrix = 104 × 104, FOV = 20.8 cm, voxel size 2 mm isotropic. Participants were asked to fixate on a white cross at the centre of a black background. Processing of images followed Human Connectome Project (HCP) minimal preprocessing pipelines.^21^

### Image analyses

#### Morphological

Processing of *T*_1_-weighted images was performed with the software Freesurfer (http://freesurfer.net) (version 5.3) using the recon-all pipeline (motion correction, non-uniform intensity normalization, Talairach transform computation, intensity normalization and skull stripping).^22-24^ Freesurer’s statistical tool Query, Design, Estimate, Contrast (QDEC) was used for whole cortex vertex-wise analysis of cortical surface, which applies the General Linear Model at each vertex to measure the correlation of the dependent variable with each cortex morphological feature. Prior to analysis, each subject was mapped to a standardized vertex space defined by the fsaverage atlas. The measure of thickness was smoothed with a 10 mm full width at half maximum (FWHM) kernel, and thickness analyses were corrected for age and gender. Monte Carlo simulation was used to correct for multiple comparisons with an initial cluster-forming threshold of *P* < 0.05, creating cluster wise *P*-values fully corrected for multiple comparisons. Cortical nodes for the GT analysis were based on the Desikan–Killiany probabilistic atlas from Freesurfer, and subcortical and cerebellar volumes were based on the probabilistic atlas from Freesurfer. Matrices were calculated based on the partial correlations between node volumes controlling for ICV.

#### Resting state

MRI images were processed using the HCP minimal processing pipelines,^21^ which is primarily based on Freesurfer and Functional MRI of the brain Software Library (FSL).^25,26^ Details on the HCP processing pipelines can be found in Glasser *et al.*^21^ In short, it performs non-linear spatial distortions removal, volumes realignment, registration to structural images, reduction of field bias, 4D image normalization to a global mean, data masking with the final brain mask and voxel mapping within the cortical grey matter ribbon onto the native cortical surface space. Additional preprocessing [motion regression using 12 motion parameters, regression-based removal of signal changes in the white matter, cerebrospinal fluid (CSF), global signal and band-pass filtering (0.01–0.1 Hz)] was performed using Analysis of Functional NeuroImages (AFNI).^27^ The combination of preprocessing pipelines recommended by the HCP, which included frame-wise registration to the single-band reference image to correct for head motion and regressing of the 12 motion parameters,^28,29^ was implemented similar to our previous work.^30^ Time-series data from four 5-min rs-fMRI scans acquired in a single session were concatenated. Three hundred and sixty time-series from Glasser Parcellation,^31^ plus 19 Freesurfer subcortical and cerebellar regions were extracted per subject,^25^ and pairwise Pearson correlations between all nodes time series (i.e. 379) were calculated and Fisher *Z*-transformed for generation of connectivity matrices.

#### Clinical, demographic and behavioural analytics

First, group comparisons across the ASEBA DSM-oriented syndrome scales were conducted by multivariate analysis of variance (MANOVA) with Šidák correction for multiple comparisons. Targeted group comparisons were conducted for other continuous variables including neuropsychological test scores, continuous sociodemographic characteristics (e.g. age, years of participant and maternal and paternal education) and concurrent validity measures that included patients’ ratings of their quality of life (QOL) in Epilepsy Inventory (QOLIE-31-P) and metrics of behavioural status [National Institutes of Health (NIH) Toolbox Emotion Battery]. Second, chi-squared tests were used for analysis of dichotomous variables (e.g. handedness, gender, mono versus polytherapy and TLE laterality).

### NIH Toolbox Emotion Battery

From the NIH Toolbox Emotion Battery for ages 18+,^32,33^ 10 subtests were selected from the dimensions of negative affect (anger affect, anger hostility, anger-physical aggression, fear affect, fear somatic arousal and sadness), psychological well-being (general life satisfaction), stress and self-efficacy (perceived stress and self-efficacy) and social relationships (loneliness). Details of item content per scale are available at https://www.healthmeasures.net/explore-measurement-systems/nih-toolbox/intro-to-nih-toolbox/emotion. Each item is responded to on a 5-point Likert scale with responses ranging from ‘not at all’ to ‘very much’ based on the past 7 days. All scales are converted to standardized *T*-scores (50 representing the mean of the US general population based on the 2010 Census).

### Quality of life (QOLIE-31-P)

The QOLIE-31-P (Patient-Weighted Quality of Life in Epilepsy Questionnaire) is an adaptation of the original QOLIE-31, a survey of health-related QOL for adults (18 years or older) with epilepsy that assesses energy (tiredness), emotions (mood), daily activities (work, driving and social), mental activity (thinking, concentrating and memory), medication effects (physical and mental), seizure worry (impact of seizures) and overall QOL .^34^

### Cognition

The ECP cognitive battery included tests from the NIH Toolbox Cognition Battery,^35^ assessing language (Oral Reading Recognition Test and Picture Vocabulary Test), attention (Flanker Inhibitory Control and Attention Test), processing speed (Pattern Comparison Processing Speed Test), executive function (List Sorting Working Memory Test) and memory (Picture Sequence Memory Test). Age-adjusted standard scores were used for analysis.

### GT matrices

The GT analysis on cortical, subcortical and cerebellar volumes (i.e. morphological matrices) was comprised of symmetric matrices of 87 nodes. Functional matrices based on temporal correlations between brain regions were constructed for each participant, and although negative correlations are intrinsically included in the fMRI data, such were removed prior to GT analyses to avoid any confounds and to have a straightforward interpretation of results.

In order to discern group differences, functional and morphological matrices were proportionally thresholded, therefore ensuring that only the strongest (highest weighted) percentage of links forms the graph, and it was combined with the minimum spanning tree (MST) as its backbone (see Garcia-Ramos *et al*.^36^ for details). For the remainder of this manuscript, each graph threshold from the morphological and functional analyses represents a combination of MST and proportional thresholding, indexed by the density level. After graph thresholding, we ensured that no graph contained negative links in order to maintain straightforward interpretations. In this study, GT metrics were calculated over a range of graph connectivity densities from 10% to 40% in 2% increments. Supplemental Tables 1 and 2 contain the list of the 87 nodes used for the morphological analysis and the 379 nodes used for the functional analysis, respectively.

### GT measures and statistical analysis

GT provides a method to investigate the topological properties of the identified phenotypes and determine how they compare with healthy controls. It provides another level of analysis in which global properties and nodal configuration can be explored further in order to derive a better understanding of overall group differences and deviation from normality. The GT measures calculated included normalized average clustering coefficient (CC), normalized global efficiency and modularity index using the MATLAB-based Brain Connectivity Toolbox (BCT) (http://www.brain-connectivity-toolbox.net/). Average CC characterizes the level of segregation of the network, global efficiency examines network integration,^37^ and modularity is a property of the network that demonstrates the presence of sub-networks or ‘modules’, with the modularity index speaking about how easily such modules are identified by the algorithm.^38^ Given that the level of segregation and integration in a graph uses a random network as a baseline for comparison,^39^ we calculated normalized versions of average CC and global efficiency. This was performed by calculating random matrices with the same number of nodes and degree distribution as the pertinent graphs and calculating the same GT measures on them. Then, GT measures (i.e. average CC and global efficiency) were divided by the same measure calculated on random matrices.

To statistically investigate group differences in the morphological analysis, each group matrix was resampled by replacement (i.e. bootstrapped) a total of 250 times. Since results from GT measures can occur by chance, each graph measure was calculated on 250 random matrices with the same number of nodes and degree distribution as the pertinent graphs. In this way, the null hypothesis could be tested. *P*-values were corrected for multiple comparisons for each of the GT measures. Since the functional analysis renders a matrix per participant, Student’s *t*-tests were performed between the actual matrices from groups.

## Results

### Participants

Table 1 provides information regarding the baseline characteristics of the control and overall TLE groups (Columns 2 and 3) as well as details of the identified cluster groups (Columns 4–6) that will be later detailed. Pairwise contrasts between controls and the overall TLE group revealed significant differences for estimated FSIQ (*P* < 0.001), age (*P* < 0.001), education (*P* = 0.003), maternal education (*P* = 0.007) and paternal education (*P* = 0.035). Gender (χ^2^ = 3.48, *df* = 3, *P* = 0.324) and handedness (χ^2^ = 10.9, *df* = 6, *P* = 0.09) did not differ significantly between the overall TLE and control groups.

**Table 1:** Sociodemographic and clinical characteristics of participants

	Controls (*n* = 83)	TLE (*n* = 114)	Cluster 1 (*n* = 42)	Cluster 2 (*n* = 48)	Cluster 3 (*n* = 24)
Age (SD)	33.8 (10.7)	39.8 (11.7)	40.05 (11.2)	40.4 (12.3)	38.33 (11.7)
Education years (SD)	15.8 (2.7)	14.7 (2.7)	15.5 (2.7)	14.3 (2.8)	14.0 (2.3)
Handedness (% RH)	90.4%	87%	88.1%	89.6%	79.2%
Gender (% female)	57%	61%	69%	51%	67%
Mother’s education years (SD)	14.6 (2.7)	13.5 (2.7)	13.6 (2.8)	13.5 (2.98)	13.4 (1.06)
Father’s education years (SD)	14.8 (2.9)	13.8 (2.9)	14.2 (3.4)	13.9 (2.7)	13.1 (2.5)
Full-scale IQ (SD)	109.9 (15.3)	100.03 (13.9)	103.2 (13.4)	97.8 (13.5)	98.9 (15.3)
Age of onset (SD)		22.5 (13.7)	23.9 (13.99)	23.2 (14.1)	19.0 (12.4)
Epilepsy duration: years (SD)		17.3 (14.3)	16.2 (13.3)	17.2 (14.6)	19.3 15.6)
ASM count		1.85 (0.94)	1.6 (0.80)	2.0 (1.0)	2.0 (1.0)
Laterality					
Left		62%	71%	59.5%	52.6%
Right		28%	21%	32.4%	26.3%
Bilateral		11%	7.9%	8.1%	10.6%

ASM, anti-seizure medication; RH, right-handed; SD, standard deviation.

### Control versus TLE participants

**Figure 1:**
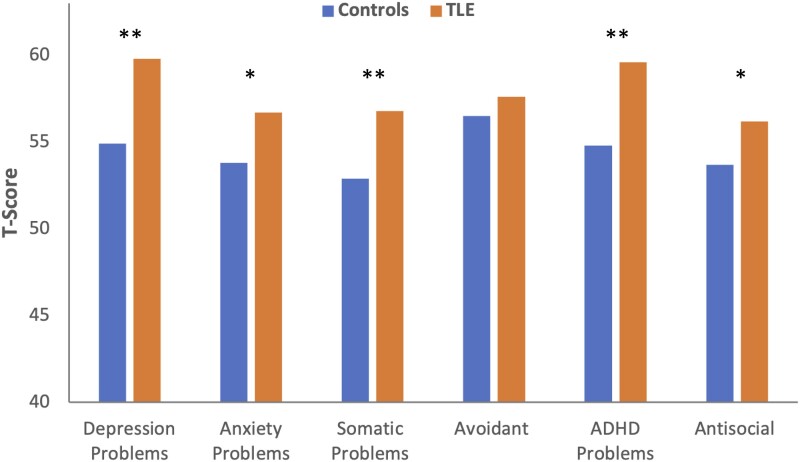
**Mean control and TLE scores for the DSM syndrome scales.** Higher scores represent greater abnormality. MANOVA was significant, Hotelling’s *T* = 0.155, *F* = 4.93, *df* = 6,191, *P* < 0.001. Student’s *t*-tests between TLE (*n* = 114) and controls (*n* = 83) revealed significant differences in all scales except avoidant (*P* = 0.386). *Significantly different at *P* < 0.05; **significantly different at *P* < 0.001.

Figure 1 shows the mean scores for the DSM-oriented scores for the TLE and control groups. MANOVA was significant, Hotelling’s *T* = 0.155, *F* = 4.93, *df* = 6,191, *P* < 0.001. Univariate effects revealed significantly greater psychopathology in the TLE group across all scales except avoidant (*P* = 0.386), with *F-*values ranging from 6.12 (antisocial) to 17.67 (somatic problems), all *P*’s < 0.001 except anxiety (*P* = 0.005) and antisocial (*P* = 0.014).

### Cluster analysis and identified latent groups

The optimal agglomerative coefficient was found using the Ward method (0.95), which was then used for hierarchical clustering. Comparable results across other methods were average (0.80), single (0.77) and complete (0.89). The optimal number of clusters determined by the Gap Statistic method was 3 (dendrogram and cluster plot provided in Supplemental Figs 1 and 2, respectively). To ensure cluster stability, several methods were employed. First K-means clustering was performed as a confirmatory test to the agglomerative hierarchal clustering on normalized behavioural data using the gap statistic method and the optimal solution was again three clusters. (Supplemental Fig. 4). There was 94.7% concordance between clusters (Supplemental Table 3) derived from K-means and hierarchical methods. Graphical evaluation of the quality of clustering was done by plotting clusters on axis derived from Principal Component 1 and Principal Component 2 seen in Supplemental Figs 3 and 5. Additionally, a bootstrap analysis (*R* = 500) was performed on the hierarchical clusters. The evaluation metric was the mean Jaccard Coefficient with the following values 0.94, 0.75 and 0.74, consistent with stable clusters.

**Figure 2:**
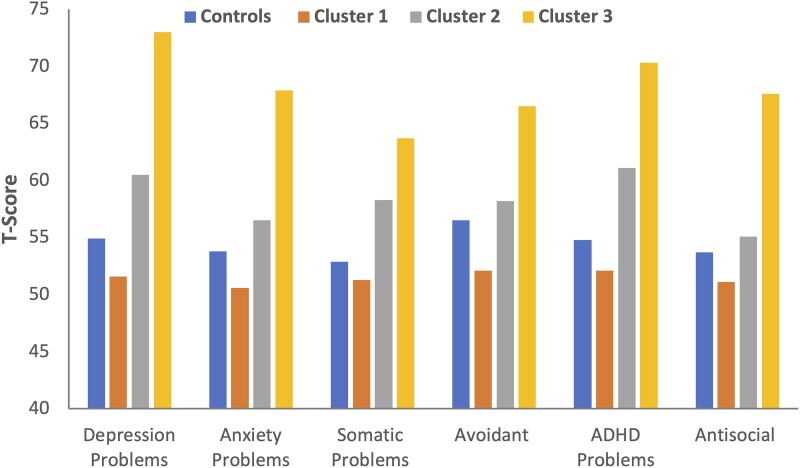
**Mean ASR DSM syndrome scale scores for the controls and TLE phenotype groups.** Higher scores represent greater abnormality. The MANOVA test comparing controls (*n* = 83) with cluster groups (*n* = 114 in total) was significant, Hotelling’s *T* = 2.37, *F* = 24.6, *df* = 18,560, *P* < 0.001. Student’s *t*-test for each ASEBA DSM-oriented scale revealed significant differences between groups with all *P*’s < 0.001.

Figure 2 provides the mean scores for the controls and the three TLE cluster groups. Comparing controls with cluster groups across scales, the MANOVA was significant, Hotelling’s *T* = 2.37, *F* = 24.6, *df* = 18,560, *P* < 0.001. Significant univariate effects were obtained for each ASEBA DSM-oriented scale with *F*’s ranging from 18.17 (avoidant) to 76.6 (depression), with all *P*’s < 0.001. Pairwise comparisons (Šidák corrected) showed the following: (i) Cluster 1 (no behavioural symptoms, 37% of TLE sample) exhibited scores in the average range across all ASEBA DSM-oriented scales, not elevated (problematic) compared with controls on any scale; (ii) Cluster 2 (mild behavioural symptoms, 42% of sample, scale scores ranging from 70th to 84th percentiles) exhibited significantly elevated ASEBA DSM-oriented scale scores compared with controls across all scales except avoidant (*P* = 0.79) and antisocial (*P* = 0.57), with significant differences on all other scales [*P*’s ranging from 0.03 (anxiety) to *P* < 0.001 (all other scales)]; (iii) Cluster 3 (severe behavioural symptoms, 21% of sample, scale scores ranging from 92nd to 99th percentiles) exhibited significantly elevated scores compared with controls across all ASEBA DSM-oriented scales (all *P*’s ≤ 0.002); Group 3 was elevated compared with Group 2 across all scales at *P* < 0.001 with the exception of somatic (*P* = 0.001) and was significantly elevated compared with Group 1 across all scales (all *P*’s < 0.001).

### NIH Toolbox Emotion Battery

**Figure 3:**
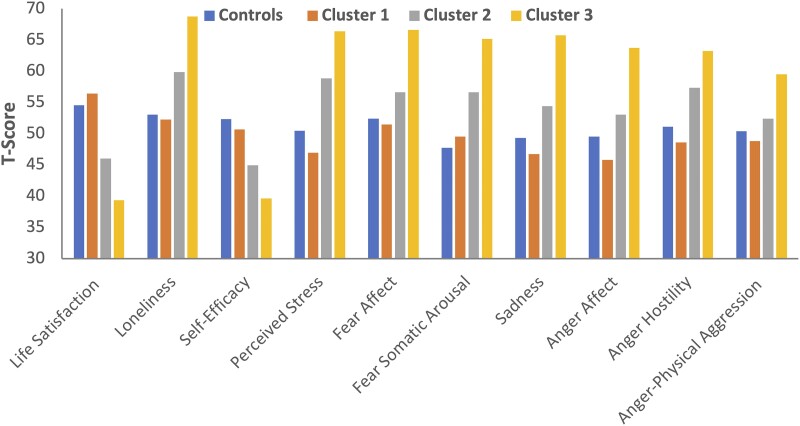
**Mean control and TLE behavioural phenotype scores on the NIH ToolBox Emotion Battery.** MANOVA was significant, Hotelling’s *T* = 1.368, *F* = 6.097, *df* = 30,401, *P* < 0.001. Each NIH Toolbox behavioural score was significantly different between groups (*n* = 83 in controls, *n* = 42 in Cluster 1, *n* = 48 in Cluster 2 and *n* = 24 in Cluster 3) with all *P*’s < 0.001.

Figure 3 provides the mean NIH Toolbox behavioural scores across the cluster groups. MANOVA was significant, Hotelling’s *T* = 1.368, *F* = 6.097, *df* = 30,401, *P* < 0.001. Univariate effects were significant across all scales ranging from *F* = 5.334 (anger-physical aggression) to *F* = 27.887 (life satisfaction). Pairwise comparisons (Šidák corrected) follow below.

Compared with controls, Cluster 1 did not differ on any scale (all *P*’s > 0.39). Compared with controls, Cluster 2 showed significantly lower (worse) scores on life satisfaction (*P* < 0.001) and self-efficacy (*P* = 0.001), with higher (worse) scores on loneliness (*P* = 0.008), perceived stress (*P* < 0.001), fear somatic (*P* < 0.001) and anger hostility (*P* = 0.017). No significant differences were seen on sadness (*P* = 0.12), anger affect (*P* = 0.44), anger-physical aggression (*P* = 0.96) and fear affect (*P* = 0.19). Compared with controls, Cluster 3 showed significantly poorer scores across all scales ranging from 0.01 (anger-physical aggression) to <0.001 across the remaining scales.

Compared with Cluster 1, Cluster 3 performed significantly worse across all scales at *P* ≤ 0.001. Compared with Cluster 1, Cluster 2 scored significantly lower on all scales but two, fear affect (*P* = 0.19) and anger-physical aggression (*P* = 0.96). Cluster 2 was significantly lower on loneliness (*P* = 0.001), self-efficacy (*P* = 0.012), fear somatic (*P* = 0.002), anger affect (*P* = 0.002), sadness (*P* = 0.002) and life satisfaction, personal stress and anger hostility (all *P*’s 0.001).

### Quality of life

**Figure 4:**
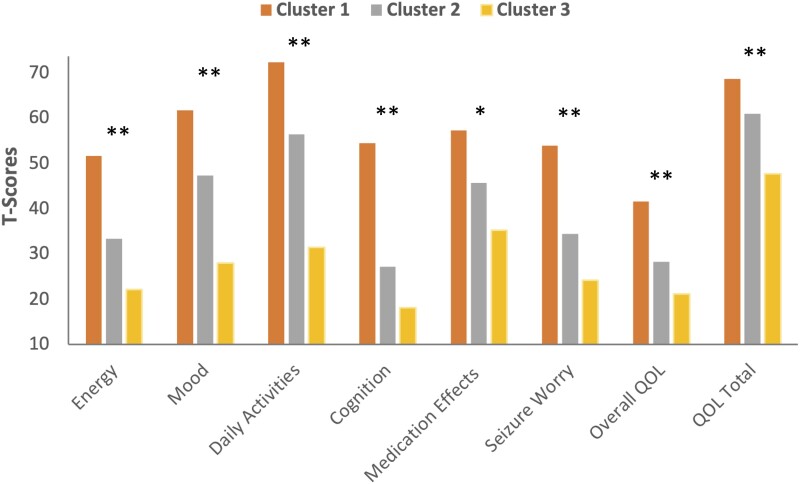
**Mean quality of life scale scores (QOLIE-31-P) for the TLE behavioural phenotype groups.** Lower scores represent greater abnormality. MANOVA was significant, Hotelling’s *T* = 0.984, *F* = 6.27, *df* = 16,204, *P* < 0.001. Student’s *t*-test for each QOLIE-31-P scale score were significantly different between groups (*n* = 42 in Cluster 1, *n* = 48 in Cluster 2 and *n* = 24 in Cluster 3). *Significantly different at *P* < 0.05; **significantly different at *P* < 0.001.

Figure 4 shows mean QOLIE-31-P scale scores across the epilepsy cluster groups. MANOVA was significant, Hotelling’s *T* = 0.984, *F* = 6.27, *df* = 16,204, *P* < 0.001. Univariate effects were significant across all QOLIE-31 scales, ranging from *P* = 0.01 for medication effects to *P* < 0.001 for all other scales, with *F*’s ranging from 4.72 (medication effects) to *F* = 26.19 (QOLIE total).

Comparison of cluster groups (with Šidák correction) showed Cluster 1 to be significantly higher (better QOL) than Cluster 2 across all scales except medication effects (*P* = 0.57) and significantly higher (better) across all scales compared with Cluster 3 with *P*’s ranging from *P* < 0.04 to *P* < 0.001. Comparison of Cluster 2 with Cluster 3 revealed significant effects for mood (*P* < 0.001), daily activities (*P* = 0.005) and QOLIE total (*P* < 0.001), without differences on energy (*P* = 0.07), cognition (*P* = 0.352), medication effects (*P* = 0.388), seizure worry (*P* = 0.389) or overall QOL (*P* = 0.098).

### Cognition

There were no effects of epilepsy behavioural cluster on the Oral Reading Recognition Test (*P* = 0.58), Picture Vocabulary Test (*P* = 0.53), Flanker Inhibitory Control and Attention Test (*P* = 0.23), List Sorting Working Memory Test (*P* = 0.86) and Picture Sequence Memory Test (*P* = 0.22). There was a significant effect of cluster for the Pattern Comparison Processing Speed Test (*P* = 0.017), where Cluster 1 was significantly faster than Cluster 3 (*P* = 0.021) with no other pairwise differences.

### Sociodemographic and clinical epilepsy characteristics

Table 1 provides the sociodemographic and clinical characteristics of the three TLE cluster groups. Among the epilepsy participants, there was no relationship between cluster membership and age (*P* = 0.78), family history of epilepsy (*P* = 0.41), gender (*P* = 0.24), race (*P* = 0.12), maternal education (*P* = 0.97) or paternal education (*P* = 0.39). The distribution of handedness was significantly different (*P* = 0.041) with a greater rate of non-right handedness in Cluster 3 (20.9%) compared with Clusters 1 (11%) and 2 (10.4%). Participant education was significantly different (*P* = 0.05) with modestly higher education in Cluster 1 (15.5 years) compared with lower levels in Clusters 2 and 3 (14.3 and 14.0 years).

Among the epilepsy participants, there was no relationship between cluster membership and age of recurrent seizure onset (*P* = 0.357), age at first seizure (*P* = 0.299), ASM (anti-seizure medication) count (*P* = 0.60), age of onset of ASM treatment (*P* = 0.61) or EEG laterality (*χ*2 = 4.1, *df* = 4, *P* = 0.34). While not reaching statistical significance, there was a pattern between cluster membership and presence of secondarily generalized tonic–clinic seizures (42.9%, 45.8% and 66.7% of participants across Clusters 1–3, *P* = 0.15) and an overall greater lifetime number of secondarily generalized seizures (19%, 29.2% and 43.4% across Clusters 1–3) (*P* = 0.12) (0–1 versus 2+).

### Cortical thickness

**Figure 5:**
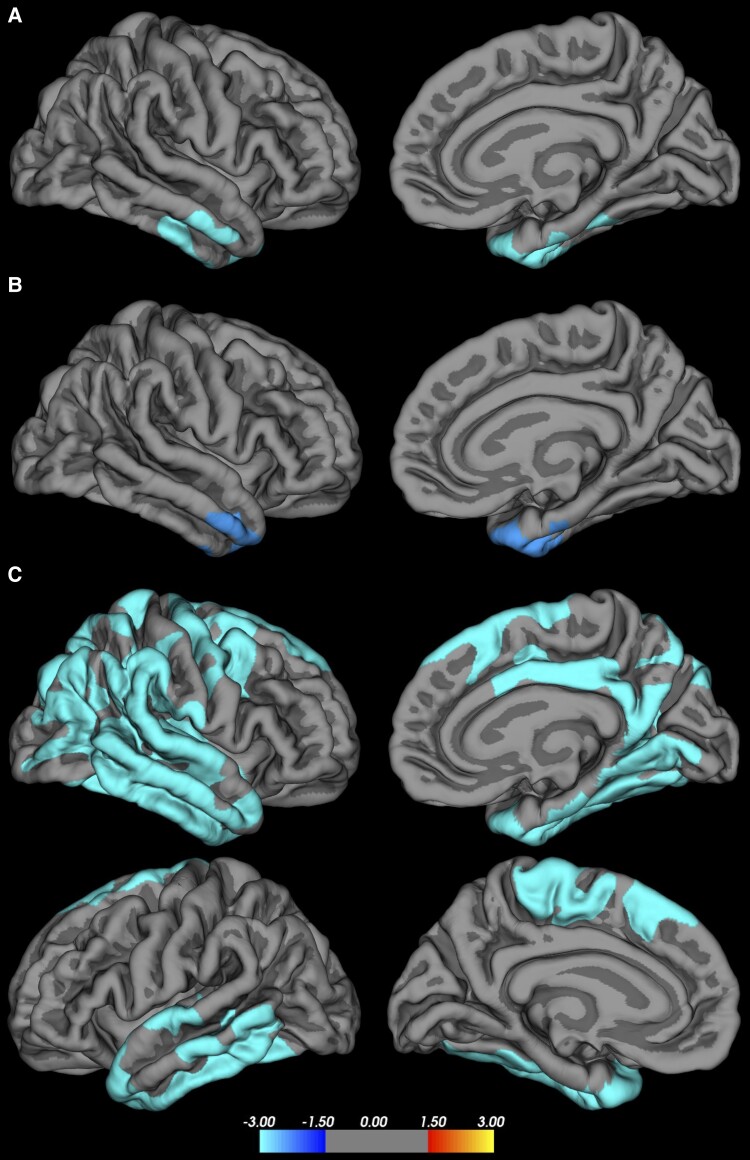
**Cortical thickness between TLE behavioural phenotypes and controls.** (**A**) Cluster 1 (*n* = 27) versus controls (*n* = 36): includes right entorhinal, middle and inferior temporal lobe (*P* < 0.001); (**B**) Cluster 2 (*n* = 24) versus controls: includes right entorhinal, middle and inferior temporal lobe (*P* < 0.001); (**C**) Cluster 3 (*n* = 11) versus controls: involvement of the right (top) and left (bottom) hemisphere including bilateral medial and lateral temporal cortex, entorhinal cortex and some involvement of cingulate, motor and pre-motor areas to a greater extent on the right that expands to parietal and occipital regions (*P* < 0.001). The scale bar indicates the *T*-value. The areas reported -represent thinner cortex in TLE compared with controls.

As depicted in Figure 5, for Clusters 1 and 2 (unimpaired and mild impairment), the regions of cortical thinning compared with controls were limited to the mesial and lateral right temporal region, primarily in the anterior entorhinal, middle and inferior temporal lobes—regions implicated in the ictogenic areas associated with TLE, comparable regions in the left temporal lobe not surviving correction. Cluster 3 shows a more extensive area of cortical thinning beyond the typical regions associated with seizure generation in TLE. First, there is an expansion of the region to include greater posterior and superior involvement of the temporal lobes on both the right and left, extending back to the temporal-occipital junction posteriorly and involving the superior temporal gyrus. In Cluster 3, there was a greater involvement of the right hemispheric cortical thinning compared with Clusters 1 and 2 with involvement of the posterior cingulate as well as parietal, temporal/parietal association areas and pre-motor regions. On the left, there was involvement of the pre-motor area and motor areas.

### GT analyses

#### Morphological connectivity

**Figure 6:**
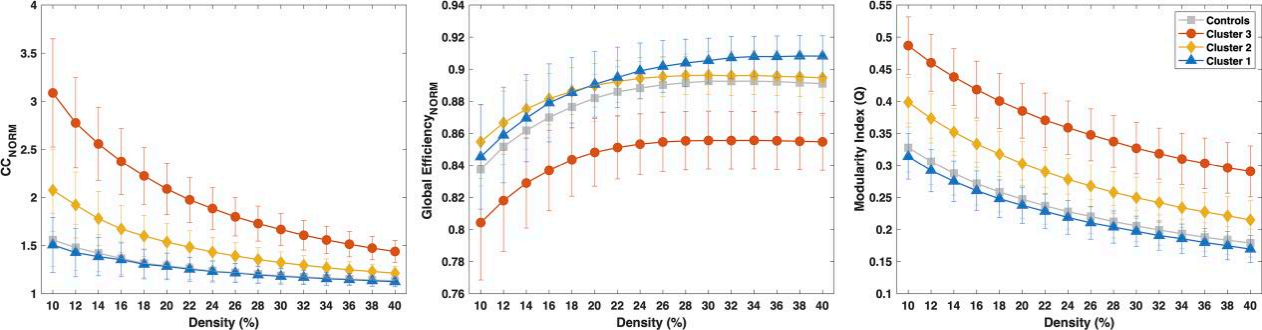
**GT measures for the morphological matrices.** Normalized average CC (left), normalized global efficiency (middle) and modularity index (right) for the whole brain on healthy controls (*n* = 36) (squares), and the three Behavioural clusters: Cluster 1 (*n* = 27) (trangles), Cluster 2 (*n* = 24) (diamonds) and Cluster 3 (*n* = 11) (circles) on morphological matrices. Student’s *t*-test showed that both Clusters 2 and 3 were significantly different from controls for normalized CC and modularity index and only Cluster 3 for normalized global efficiency, in which the other two clusters were significantly different from controls for a range of density values (Cluster 1 from 24% to 40% and Cluster 2 at 10%; *P* < 0.001, FDR corrected).

Three global GT metrics were assessed based on morphological connectivity: normalized CC, global efficiency and modularity index (Fig. 6). The following analysis included 36 controls, 27 TLE participants in Cluster 1 (unimpaired), 24 in Cluster 2 (mild) and 11 in Cluster 3 (severe). In both the normalized CC and modularity, there was a stepwise progression from controls and ‘unimpaired’ TLE phenotype to the ‘severe’ phenotype, with the severe phenotype having a higher CC and modularity. These results reflect the extended pattern of cortical thinning in the epileptogenic zone and associated regions (Fig. 5). These linked atrophic regions create a higher morphological correlation coefficient that results in increased overall CC and modularity. Student’s *t*-tests showed that Cluster 1 (‘unimpaired’) was not significantly different compared with controls for any of the densities for both normalized CC and modularity index (*P* > 0.05). However, both the moderate and severe clusters were significantly different from controls for each density level and for both normalized CC and modularity index (*P* < 0.001). The global efficiency is decreased within the severely impaired group, while there is less separation between the other groups. Student’s *t*-tests showed that the ‘unimpaired’ group showed significant differences compared with controls for a range of densities (24–40%, *P* < 0.001) and the group with ‘moderate’ impairment for 10% (*P* < 0.001). However, the severe phenotype showed significant differences for each density value compared with controls (*P* < 0.001). Again, these findings reflect the separation of the epileptogenic and associated regions of atrophy from the rest of the brain.

#### Resting-state functional MRI

**Figure 7:**
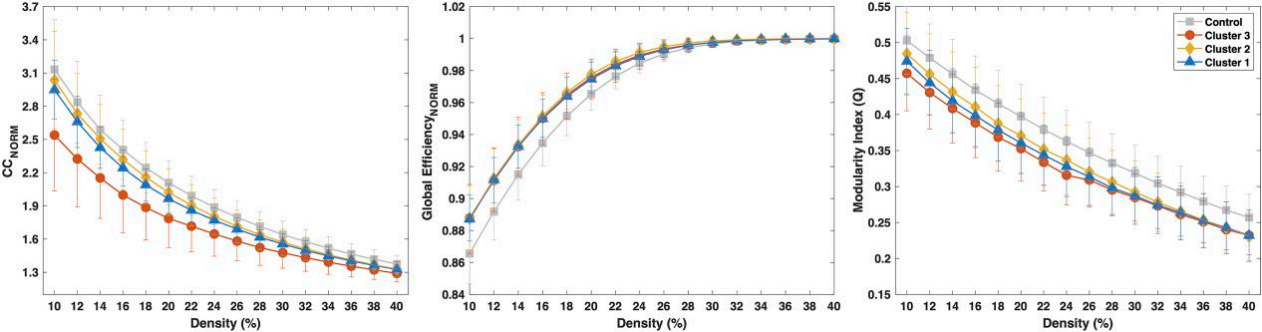
**GT measures for the functional matrices.** Normalized average CC (left), normalized global efficiency (middle) and modularity index (right) on healthy controls (*n* = 36) (squares) and the three Behavioural clusters: Cluster 1 (*n* = 27) (triangles), Cluster 2 (*n* = 23) (diamonds) and Cluster 3 (*n* = 11) (circles) on functional matrices. Student’s *t*-test showed that none of the clusters were significantly different from controls (*P* > 0.05).

In this analysis, participants with rs-fMRI included the following: controls (*n* = 36), Cluster 1 (*n* = 27), Cluster 2 (*n* = 23) and Cluster 3 (*n* = 11). Using the same global GT metrics as in the morphological analysis, the results using functional data overall show less separation between groups than the morphological GT (Fig. 7), and the results point in the opposite direction. The normalized CC is lower in the ‘severely impaired’ group. The global efficiency is increased in all the TLE participants relative to the controls, and the modularity is decreased in all TLE participants but to the greatest extent in the ‘severely impaired’ group.

## Discussion

This application of unsupervised machine learning procedures addressed and characterized the underlying heterogeneity of psychopathology in TLE and the sociodemographic, clinical and neuroimaging correlates of the identified taxonomy. Five core findings were revealed.

First, congruent with the existing literature, TLE participants, as a group, reported more psychopathology compared with healthy controls with significantly higher (abnormal) scores across all ASEBA DSM-oriented scales (Fig. 1). While statistically significant, the elevations in the TLE group were mild in degree and did not reach the ASEBA-defined borderline impaired range of clinical abnormality—arguably a grey zone of anomaly (statistically but not clinically notable). But this increased risk of psychopathology and self-reported distress is a well-appreciated pattern and has long coloured the view of the behaviour risk associated with this epilepsy syndrome.

Second, embedded within the mean profile described above were three distinct latent groups that varied significantly in their pattern of psychological risk (Fig. 2), ranging from equivalent (or less) risk compared with controls (Cluster 1), to clinically meaningful psychopathology (Cluster 3), generally exceeding the 94th percentile across the individual scales (with the exception of somatic problems), and an intermediate and mildly impacted group (Cluster 2). The person-centred analytic approach undertaken here underscores the heterogeneity of behavioural risk among patients with TLE and resulted in a more clinically meaningful separation of groups than the tradition TLE versus control comparison. Only two other investigations have taken this approach.^15,16^ In an investigation of adults with TLE (*n* = 96) and controls (*n* = 82) using a different measure of psychopathology (Symptom Checklist 90-Revised),^15^ the behavioural results were similar to this investigation in that (i) TLE patients as a group demonstrated significantly greater psychopathology across the behavioural measures compared with controls in both investigations; (ii) three underlying behavioural clusters were identified reflecting stepwise increases in psychopathology with a cluster characterized by comparable or better performance compared with controls, a severely affected group and an intermediate group, with proportions equivalent to those reported here; (iii) cluster membership was associated with (different) indicators of validity [Structured Clinical Interview for DSM Disorders (SCID) diagnoses in the prior investigation and NIH Toolbox behavioural measures here and QOL metrics in both investigations]; and (iv) both studies identified demographic, clinical and imaging metrics associated with the clusters. A recent investigation of 81 children with TLE (*n* = 81) using the Behavioural Assessment System for Children-2 also demonstrated three behavioural phenotypes including no behavioural concerns (43%), internalizing (16%) and externalizing problems (41%), again associated with clinical variables, psychosocial and familial factors, everyday executive function and QOL.^16^ Thus, the phenotype approach appears reliable and reproducible across studies and robust in its ability to identify meaningful behavioural subgroups with applicability to both children and adults with epilepsy.

Third, concurrent validity of the latent groups identified here was demonstrated through their association with independent measures of QOL (QOLIE-31-P) (Fig. 4) and metrics of negative affect, psychological well-being, stress and self-efficacy and social relationships (NIH Toolbox Emotion Battery) (Fig. 3). These independent measures, examined as a function of the identified behavioural phenotypes, mirrored the identical stepwise patterns of abnormality reflected in the latent groups. Clearly, the behavioural phenotypes have implications for other dimensions of behaviour and function. In this investigation, we did not have independent psychiatric diagnoses that would have been a valuable measure of concurrent validity. But prior investigations have shown that identified behavioural clusters do have external validity demonstrated through their relationship with formal psychiatric assessments.

Fourth, examination of sociodemographic, clinical and cognitive measures demonstrated relationships with the behavioural clusters. The most impaired behavioural phenotype (Cluster 3) was associated with the highest rate of left-handedness and lower education. Cognitive correlates were quite modest, but the greatest degree of cognitive slowing was seen in Cluster 3.

Fifth, analyses of cortical thickness provide another external marker of concurrent validity and suggest a potential pathogenic substrate contributing to the behavioural differences (Fig. 5). The normal and mildly symptomatic groups (Clusters 1 and 2) exhibit a modest extent of cortical thinning, limited to the anterior and mesial regions of the temporal lobe, predominantly on the right. The severely abnormal group (Cluster 3) was distinct with cortical thinning throughout the bilateral temporal lobes and extending into the limbic seizure network through the right posterior cingulate and mesial parietal regions as well the temporal/parietal association areas. Further, the pre-motor and motor areas were involved on both the right and left. The involvement of these motor/pre-motor areas may be related to the greater propensity for these patients to experience secondarily generalized seizures and supports the hypothesis that even in focal epilepsy, the primary or secondary effects of the seizure generating regions may have pathogenic downstream consequences with a meaningful global effect on cognition and behaviour. The distributed morphological findings in the severely abnormal cluster also represent a direct challenge to the classic view that focal medial temporal lobe pathology is associated with psychopathological risk—in fact the opposite was true.

The morphology-based GT analysis (Fig. 6) reinforces the results of the cortical thickness findings. The CC and modularity were both increased in the severe (Cluster 3) group and demonstrated a step-wise march towards the controls as a function of the behavioural phenotypes. These results can be explained by a network of atrophy extending along the epileptogenic zone and areas that are functionally connected (such as pre-motor, motor, parietal and posterior cingulate). The regions of atrophy had higher internal correlations that were reflected in high CC and modularity. The lack of integration of these regions of atrophy decreased the global efficiency. This lack of integration may account for some of the downstream cognitive and behavioural consequences of epilepsy.

The functional GT analyses (Fig. 7) did not exhibit the same degree of separation between groups with an overall trend that revealed lower CC, higher global efficiency and lower modularity. As expected, the functional and morphological global GT metrics had opposite directions. For example, the morphological CC was higher in the severely impaired group (Cluster 3) compared with controls in distinction to the functional analysis that had a lower CC in the severely impaired group. This pattern has been reported in other studies^40^ and may seem paradoxical at first, but, on further inspection, it creates a fuller explanation of the process by which the epileptic network disrupts cognition and behaviour. The epileptic network is highly internally connected and relatively disconnected from the rest of the brain.^40,41^ This is reflected morphologically in a group of regions that all exhibit similar degrees of cortical thinning. This network stands in contradistinction to the rest of the brain where cortical thickness remains relatively normal. Morphological GT metrics reflect this standout network of cortical atrophy and show increased modularity and CC. The functional GT is quite different. As opposed to the morphological connectivity, the functional connectivity in controls is highly organized into densely connected subnetworks (e.g. default mode network). As regions become involved in the epileptic network, they disconnect from this highly integrated modular network. In turn, this decreases the overall modularity of the functional networks and decreases the CC. It is unclear if this process of increased modularity and CC in morphologic connectivity and a decrease in functional connectivity is specific to epilepsy or if it is a more general process that can also be found in neurodegenerative disorders such as Alzheimer’s disease.

### Implications for comorbidity research

The long-standing ‘TLE-psychopathology’ issue, like other hypotheses regarding particular behavioural or cognitive comorbidities, centred predominantly on the overall behavioural risk linked to a particular epilepsy syndrome. As is case for cognition, there is significant variability in patient-presented emotional–behavioural distress. The move to identify underlying phenotypic presentations is consistent with a more individualized precision-based approach to understanding the presence, nature and distribution of problematic comorbidities. This project focused on TLE and research of this type needs to be expanded to other epilepsy syndromes to determine the degree to which behavioural clusters may cut across other epilepsies. Such research would inform a taxonomy of the comorbidities of epilepsy and the specificity of their linkage to traditional epilepsy classifications.

More generally, these behavioural findings, especially when considered in the broader context of cognitive phenotype investigations, are congruent with the hypothesis that the heterogeneity in the neurobehavioural status of patents with TLE can be captured and validated by unsupervised machine learning approaches representing a more nuanced view of the comorbidity risks of epilepsy and its taxonomy. Notable is the significant proportion of patients largely comparable with controls—a group rarely emphasized, a mildly abnormal, and the third and minority severely problematic in cognition and behaviour. Discrete behavioural phenotypes, detected in adults and children, are derived from clinical settings, and it is likely that comparable investigations in population-based cohorts would provide more accurate estimates of phenotype proportions.

## Supplementary Material

fcad095_Supplementary_Data

## Data Availability

The data that support the findings of this study are available from the corresponding author, upon reasonable request.

## Abbreviations

AFNI =: Analysis of Functional NeuroImages
ASEBA =: Achenbach System of Empirically Based Assessment
ASM =: anti-seizure medication
BCT =: Brain Connectivity Toolbox
CC =: clustering coefficient
CSF =: cerebrospinal fluid
DSM =: Diagnostic and Statistical Manual of Mental Disorders
DWI =: diffusion-weighted imaging
ECP =: Epilepsy Connectome Project
EMU =: epilepsy monitoring unit
FDR =: false discovery rate
FSL =: Functional MRI of the brain Software Library
FWHM =: full width at half maximum
FSIQ =: full-scale intelligence quotient
GT =: graph theory
ICD =: International Classification of Diseases
ICV =: intracranial volume
HCP =: Human Connectome Project
MANOVA =: multivariate analysis of variance
MST =: minimum spanning tree
NIH =: National Institutes of Health
QDEC =: Query, Design, Estimate, Contrast
QOL =: quality of life
QOLIE =: Quality of Life in Epilepsy Inventory
RH =: right-handed
rs-fMRI =: resting-state functional MRI
SCID =: Structured Clinical Interview for DSM Disorders
SD =: standard deviation
TLE =: temporal lobe epilepsy
*T*-score =: hypothesis test statistic/score
