# Supplementary material for: The presence, nature and network characteristics of behavioural phenotypes in temporal lobe epilepsy
Source: Brain Commun. 2023 Mar 30;5(2):fcad095. doi: 10.1093/braincomms/fcad095 (PMC10082555; doi:10.1093/braincomms/fcad095)
Supplement: fcad095_Supplementary_Data [file fcad095_supplementary_data.pdf]

Supplemental Table 1: Morphological Nodes

	Node	Abbreviation
1	lh_frontalpole	L_PoleF
2	lh_superiorfrontal	L_SupF
3	lh_rostralmiddlefrontal	L_RosMidF
4	lh_caudalmiddlefrontal	L_CaudMidF
5	lh_lateralorbitofrontal	L_LatOrbF
6	lh_medialorbitofrontal	L_MedOrbF
7	lh_parsopercularis	L_ParsOp
8	lh_parsorbitalis	L_ParsOrb
9	lh_parstriangularis	L_ParsTri
10	lh_precentral	L_Precent
11	lh_insula	L_Ins
12	lh_paracentral	L_Parac
13	lh_postcentral	L_Postc
14	lh_superiorparietal	L_SupP
15	lh_precuneus	L_Precun
16	lh_inferiorparietal	L_InfP
17	lh_supramarginal	L_Supram
18	lh_temporalpole	L_PoleT
19	lh_superiortemporal	L_SupT
20	lh_middletemporal	L_MidT
21	lh_inferiortemporal	L_InfT
22	lh_bankssstsT	L_Bankssts
23	lh_entorhinalT	L_Enthorh
24	lh_transversetemporal	L_TransvT
25	lh_parahippocampal	L_Parahipp
26	lh_fusiform	L_Fusif
27	lh_cuneus	L_Cuneus
28	lh_lateraloccipital	L_LatO
29	lh_lingual	L_Ling
30	lh_pericalcarine	L_Pericalc
31	lh_rostralanteriorcingulate	L_RosAntC
32	lh_caudalanteriorcingulate	L_CaudAntC
33	lh_isthmuscingulate	L_IsthmC
34	lh_posteriorcingulate	L_PostC
35	rh_frontalpole	R_PoleF
36	rh_superiorfrontal	R_SupF
37	rh_rostralmiddlefrontal	R_RosMidF
38	rh_caudalmiddlefrontal	R_CaudMidF
39	rh_lateralorbitofrontal	R_LatOrbF
40	rh_medialorbitofrontal	R_MedOrbF
41	rh_parsopercularis	R_ParsOp

42	rh_parsorbitalis	R_ParsOrb
43	rh_parstriangularis	R_ParsTri
44	rh_precentral	R_Precent
45	rh_insula	R_Ins
46	rh_paracentral	R_Parac
47	rh_postcentral	R_Postc
48	rh_superiorparietal	R_SupP
49	rh_precuneus	R_Precun
50	rh_inferiorparietal	R_InfP
51	rh_supramarginal	R_Supram
52	rh_temporalpole	R_PoleT
53	rh_superiortemporal	R_SupT
54	rh_middletemporal	R_MidT
55	rh_inferiortemporal	R_InfT
56	rh_bankssts	R_Bankssts
57	rh_entorhinal	R_Enthorh
58	rh_transversetemporal	R_TransvT
59	rh_parahippocampal	R_Parahipp
60	rh_fusiform	R_Fusif
61	rh_cuneus	R_Cuneus
62	rh_lateraloccipital	R_LatO
63	rh_lingual	R_Ling
64	rh_pericalcarine	R_Pericalc
65	rh_rostralanteriorcingulate	R_RosAntC
66	rh_caudalanteriorcingulate	R_CaudAntC
67	rh_isthmuscingulate	R_IsthmC
68	rh_posteriorcingulate	R_PostC
69	Left-Thalamus	L_Thal
70	Left-Caudate	L_Caud
71	Left-Putamen	L_Putam
72	Left-Pallidum	L_Pallid
73	Left-Hippocampus	L_Hippoc
74	Left-Amygdala	L_Amyg
75	Left-Accumbens-area	L_Accumb
76	Left-VentralDC	L_VentDC
77	Left-Cerebellum	L_Cereb
78	Right-Thalamus	R_Thal
79	Right-Caudate	R_Caud
80	Right-Putamen	R_Putam
81	Right-Pallidum	R_Pallid
82	Right-Hippocampus	R_Hippoc
83	Right-Amygdala	R_Amyg
84	Right-Accumbens-area	R_Accumb

85	Right-VentralDC	R_VentDC
86	Right-Cerebellum	R_Cereb
87	Brain-Stem	BrainStem

Supplemental Table 2: Functional Nodes

Glasser Parcel Number	Glasser Node Label	Glasser Region (22 total)
1	L_Primary Visual Cortex	Primary Visual Cortex (V1)
2	L_Medial Superior Temporal Area	MT+ Complex and Neighbors
3	L_Sixth Visual Area	Dorsal Stream
4	L_Second Visual Area	Early Visual Cortex
5	L_Third Visual Area	Early Visual Cortex
6	L_Fourth Visual Area	Early Visual Cortex
7	L_Eighth Visual Area	Ventral Stream
8	L_Primary Motor Cortex	Somatosensory and Motor Cortex
9	L_Primary Sensory Cortex	Somatosensory and Motor Cortex
10	L_Frontal Eye Fields	Premotor Cortex
11	L_Premotor Eye Field	Premotor Cortex
12	L_Area 55b	Premotor Cortex
13	L_Area V3A	Dorsal Stream
14	L_RetroSplenial Complex	Posterior Cingulate Cortex
15	L_Parieto-Occipital Sulcus Area 2	Posterior Cingulate Cortex
16	L_Seventh Visual Area	Dorsal Stream
17	L_IntraParietal Sulcus Area 1	Dorsal Stream
18	L_Fusiform Face Complex	Ventral Stream
19	L_Area V3B	Dorsal Stream
20	L_Area Lateral Occipital 1	MT+ Complex and Neighbors
21	L_Area Lateral Occipital 2	MT+ Complex and Neighbors
22	L_Posterior InferoTemporalComplex	Ventral Stream
23	L_Middle Temporal Area	MT+ Complex and Neighbors
24	L_Primary Auditory Cortex	Early Auditory Cortex

25	L_PeriSylvian Language Area	Temporal-Parietal-Occipital Junction
26	L_Superior Frontal Language Area	Dorsolateral Prefrontal Cortex
27	L_PreCuneus Visual Area	Posterior Cingulate Cortex
28	L_Superior Temporal Visual Area	Temporal-Parietal-Occipital Junction
29	L_Medial Area 7P	Superior Parietal and IPS Cortex
30	L_Area 7m	Posterior Cingulate Cortex
31	L_Parieto-Occipital Sulcus Area 1	Posterior Cingulate Cortex
32	L_Area 23d	Posterior Cingulate Cortex
33	L_Area ventral 23 a+b	Posterior Cingulate Cortex
34	L_Area dorsal 23 a+b	Posterior Cingulate Cortex
35	L_Area 31p ventral	Posterior Cingulate Cortex
36	L_Area 5m	Sensorimotor Associated Paracentral Lobular and Mid Cingulate Cortex
37	L_Area 5m ventral	Sensorimotor Associated Paracentral Lobular and Mid Cingulate Cortex
38	L_Area 23c	Posterior Cingulate Cortex
39	L_Area 5L	Sensorimotor Associated Paracentral Lobular and Mid Cingulate Cortex
40	L_Dorsal Area 24d	Sensorimotor Associated Paracentral Lobular and Mid Cingulate Cortex
41	L_Ventral Area 24d	Sensorimotor Associated Paracentral Lobular and Mid Cingulate Cortex
42	L_Lateral Area 7A	Superior Parietal and IPS Cortex
43	L_Supplementary and Cingulate Eye Field	Sensorimotor Associated Paracentral Lobular and Mid Cingulate Cortex
44	L_Area 6m anterior	Sensorimotor Associated Paracentral Lobular and Mid Cingulate Cortex
45	L_Medial Area 7A	Superior Parietal and IPS Cortex
46	L_Lateral Area 7P	Superior Parietal and IPS Cortex
47	L_Area 7PC	Superior Parietal and IPS Cortex
48	L_Area Lateral IntraParietal ventral	Superior Parietal and IPS Cortex
49	L_Ventral IntraParietal Complex	Superior Parietal and IPS Cortex
50	L_Medial IntraParietal Area	Superior Parietal and IPS Cortex

51	L_Area 1	Somatosensory and Motor Cortex
52	L_Area 2	Somatosensory and Motor Cortex
53	L_Area 3a	Somatosensory and Motor Cortex
54	L_Dorsal area 6	Premotor Cortex
55	L_Area 6mp	Sensorimotor Associated Paracentral Lobular and Mid Cingulate Cortex
56	L_Ventral Area 6	Premotor Cortex
57	L_Area Posterior 24 prime	Anterior Cingulate and Medial Prefrontal Cortex
58	L_Area 33 prime	Anterior Cingulate and Medial Prefrontal Cortex
59	L_Anterior 24 prime	Anterior Cingulate and Medial Prefrontal Cortex
60	L_Area p32 prime	Anterior Cingulate and Medial Prefrontal Cortex
61	L_Area a24	Anterior Cingulate and Medial Prefrontal Cortex
62	L_Area dorsal 32	Anterior Cingulate and Medial Prefrontal Cortex
63	L_Area 8BM	Anterior Cingulate and Medial Prefrontal Cortex
64	L_Area p32	Anterior Cingulate and Medial Prefrontal Cortex
65	L_Area 10r	Anterior Cingulate and Medial Prefrontal Cortex
66	L_Area 47m	Orbital and Polar Frontal Cortex
67	L_Area 8Av	Dorsolateral Prefrontal Cortex
68	L_Area 8Ad	Dorsolateral Prefrontal Cortex
69	L_Area 9 Middle	Anterior Cingulate and Medial Prefrontal Cortex
70	L_Area 8B Lateral	Dorsolateral Prefrontal Cortex
71	L_Area 9 Posterior	Dorsolateral Prefrontal Cortex
72	L_Area 10d	Orbital and Polar Frontal Cortex
73	L_Area 8C	Dorsolateral Prefrontal Cortex
74	L_Area 44	Inferior Frontal Cortex

75	L_Area 45	Inferior Frontal Cortex
76	L_Area 47l (47 lateral)	Inferior Frontal Cortex
77	L_Area anterior 47r	Orbital and Polar Frontal Cortex
78	L_Rostral Area 6	Premotor Cortex
79	L_Area IFJa	Inferior Frontal Cortex
80	L_Area IFJp	Inferior Frontal Cortex
81	L_Area IFSp	Inferior Frontal Cortex
82	L_Area IFSa	Inferior Frontal Cortex
83	L_Area posterior 9-46v	Dorsolateral Prefrontal Cortex
84	L_Area 46	Dorsolateral Prefrontal Cortex
85	L_Area anterior 9-46v	Dorsolateral Prefrontal Cortex
86	L_Area 9-46d	Dorsolateral Prefrontal Cortex
87	L_Area 9 anterior	Dorsolateral Prefrontal Cortex
88	L_Area 10v	Anterior Cingulate and Medial Prefrontal Cortex
89	L_Area anterior 10p	Orbital and Polar Frontal Cortex
90	L_Polar 10p	Orbital and Polar Frontal Cortex
91	L_Area 11l	Orbital and Polar Frontal Cortex
92	L_Area 13l	Orbital and Polar Frontal Cortex
93	L_Orbital Frontal Complex	Orbital and Polar Frontal Cortex
94	L_Area 47s	Orbital and Polar Frontal Cortex
95	L_Area Lateral IntraParietal dorsal	Superior Parietal and IPS Cortex
96	L_Area 6 anterior	Premotor Cortex
97	L_Inferior 6-8 Transitional Area	Dorsolateral Prefrontal Cortex
98	L_Superior 6-8 Translational Area	Dorsolateral Prefrontal Cortex
99	L_Area 43	Posterior Opercular Cortex
100	L_Area OP4/PV	Posterior Opercular Cortex
101	L_Area OP1/SII	Posterior Opercular Cortex
102	L_Area OP2-3/VS	Posterior Opercular Cortex
103	L_Area 52	Insular and Frontal Opercular Cortex

104	L_RetroInsular Cortex	Early Auditory Cortex
105	L_Area PFcm	Posterior Opercular Cortex
106	L_Posterior Insular Area 2	Insular and Frontal Opercular Cortex
107	L_Area TA2	Auditory Association Cortex
108	L_Frontal OPercular Area 4	Insular and Frontal Opercular Cortex
109	L_Middle Insular Area	Insular and Frontal Opercular Cortex
110	L_Pirform Cortex	Insular and Frontal Opercular Cortex
111	L_Anterior Ventral Insular Area	Insular and Frontal Opercular Cortex
112	L_Anterior Agranular Insula Complex	Insular and Frontal Opercular Cortex
113	L_Frontal OPercular Area 1	Posterior Opercular Cortex
114	L_Frontal OPercular Area 3	Insular and Frontal Opercular Cortex
115	L_Frontal OPercular Area 2	Insular and Frontal Opercular Cortex
116	L_Area PFt	Inferior Parietal Cortex
117	L_Anterior IntraParietal Area	Superior Parietal and IPS Cortex
118	L_Entorhinal Cortex	Medial Temporal Cortex
119	L_PreSubiculum	Medial Temporal Cortex
120	L_Hippocampus	Medial Temporal Cortex
121	L_ProStriate Area	Posterior Cingulate Cortex
122	L_Perirhinal Ectorhinal Cortex	Medial Temporal Cortex
123	L_Area STGa	Auditory Association Cortex
124	L_ParaBelt Complex	Early Auditory Cortex
125	L_Auditory 5 Complex	Auditory Association Cortex
126	L_ParaHippocampal Area 1	Medial Temporal Cortex
127	L_ParaHippocampal Area 3	Medial Temporal Cortex
128	L_Area STSd anterior	Auditory Association Cortex

129	L_Area STSd posterior	Auditory Association Cortex
130	L_Area STSv posterior	Auditory Association Cortex
131	L_Area TG dorsal	Lateral Temporal Cortex
132	L_Area TE1 anterior	Lateral Temporal Cortex
133	L_Area TE1 posterior	Lateral Temporal Cortex
134	L_Area TE2 anterior	Lateral Temporal Cortex
135	L_Area TF	Lateral Temporal Cortex
136	L_Area TE2 posterior	Lateral Temporal Cortex
137	L_Area PHT	Lateral Temporal Cortex
138	L_Area PH	MT+ Complex and Neighbors
139	L_Area TemporoParietoOccipital Junction 1	Temporal-Parietal-Occipital Junction
140	L_Area TemporoParietoOccipital Junction 2	Temporal-Parietal-Occipital Junction
141	L_Area TemporoParietoOccipital Junction 3	Temporal-Parietal-Occipital Junction
142	L_Dorsal Transitional Visual Area	Posterior Cingulate Cortex
143	L_Area PGp	Inferior Parietal Cortex
144	L_Area IntraParietal 2	Inferior Parietal Cortex
145	L_Area IntraParietal 1	Inferior Parietal Cortex
146	L_Area IntraParietal 0	Inferior Parietal Cortex
147	L_Area PF opercular	Inferior Parietal Cortex
148	L_Area PF Complex	Inferior Parietal Cortex
149	L_Area PFm Complex	Inferior Parietal Cortex
150	L_Area PGi	Inferior Parietal Cortex
151	L_Area PGs	Inferior Parietal Cortex
152	L_Area V6A	Dorsal Stream
153	L_VentroMedial Visual Area 1	Ventral Stream

154	L_VentroMedial Visual Area 3	Ventral Stream
155	L_ParaHippocampal Area 2	Medial Temporal Cortex
156	L_Area V4t	MT+ Complex and Neighbors
157	L_Area FST	MT+ Complex and Neighbors
158	L_Area V3CD	MT+ Complex and Neighbors
159	L_Area Lateral Occipital 3	MT+ Complex and Neighbors
160	L_VentroMedial Visual Area 2	Ventral Stream
161	L_Area 31pd	Posterior Cingulate Cortex
162	L_Area 31a	Posterior Cingulate Cortex
163	L_Ventral Visual Complex	Ventral Stream
164	L_Area 25	Anterior Cingulate and Medial Prefrontal Cortex
165	L_Area s32	Anterior Cingulate and Medial Prefrontal Cortex
166	L_posterior OFC Complex	Orbital and Polar Frontal Cortex
167	L_Area Posterior Insular 1	Insular and Frontal Opercular Cortex
168	L_Insular Granular Complex	Insular and Frontal Opercular Cortex
169	L_Area Frontal Opercular 5	Insular and Frontal Opercular Cortex
170	L_Area posterior 10p	Orbital and Polar Frontal Cortex
171	L_Area posterior 47r	Inferior Frontal Cortex
172	L_Area TG Ventral	Lateral Temporal Cortex
173	L_Medial Belt Complex	Early Auditory Cortex
174	L_Lateral Belt Complex	Early Auditory Cortex
175	L_Auditory 4 Complex	Auditory Association Cortex
176	L_Area STSv anterior	Auditory Association Cortex
177	L_Area TE1 Middle	Lateral Temporal Cortex

178	L_Para-Insular Area	Insular and Frontal Opercular Cortex
179	L_Area anterior 32 prime	Anterior Cingulate and Medial Prefrontal Cortex
180	L_Area posterior 24	Anterior Cingulate and Medial Prefrontal Cortex
181	R_Primary Visual Cortex	Primary Visual Cortex (V1)
182	R_Medial Superior Temporal Area	MT+ Complex and Neighbors
183	R_Sixth Visual Area	Dorsal Stream
184	R_Second Visual Area	Early Visual Cortex
185	R_Third Visual Area	Early Visual Cortex
186	R_Fourth Visual Area	Early Visual Cortex
187	R_Eighth Visual Area	Ventral Stream
188	R_Primary Motor Cortex	Somatosensory and Motor Cortex
189	R_Primary Sensory Cortex	Somatosensory and Motor Cortex
190	R_Frontal Eye Fields	Premotor Cortex
191	R_Premotor Eye Field	Premotor Cortex
192	R_Area 55b	Premotor Cortex
193	R_Area V3A	Dorsal Stream
194	R_RetroSplenial Complex	Posterior Cingulate Cortex
195	R_Parieto-Occipital Sulcus Area 2	Posterior Cingulate Cortex
196	R_Seventh Visual Area	Dorsal Stream
197	R_IntraParietal Sulcus Area 1	Dorsal Stream
198	R_Fusiform Face Complex	Ventral Stream
199	R_Area V3B	Dorsal Stream
200	R_Area Lateral Occipital 1	MT+ Complex and Neighbors
201	R_Area Lateral Occipital 2	MT+ Complex and Neighbors
202	R_Posterior InferoTemporalComplex	Ventral Stream
203	R_Middle Temporal Area	MT+ Complex and Neighbors
204	R_Primary Auditory Cortex	Early Auditory Cortex
205	R_PeriSylvian Language Area	Temporal-Parietal-Occipital Junction
206	R_Superior Frontal Language Area	Dorsolateral Prefrontal Cortex

207	R_PreCuneus Visual Area	Posterior Cingulate Cortex
208	R_Superior Temporal Visual Area	Temporal-Parietal-Occipital Junction
209	R_Medial Area 7P	Superior Parietal and IPS Cortex
210	R_Area 7m	Posterior Cingulate Cortex
211	R_Parieto-Occipital Sulcus Area 1	Posterior Cingulate Cortex
212	R_Area 23d	Posterior Cingulate Cortex
213	R_Area ventral 23 a+b	Posterior Cingulate Cortex
214	R_Area dorsal 23 a+b	Posterior Cingulate Cortex
215	R_Area 31p ventral	Posterior Cingulate Cortex
216	R_Area 5m	Sensorimotor Associated Paracentral Lobular and Mid Cingulate Cortex
217	R_Area 5m ventral	Sensorimotor Associated Paracentral Lobular and Mid Cingulate Cortex
218	R_Area 23c	Posterior Cingulate Cortex
219	R_Area 5L	Sensorimotor Associated Paracentral Lobular and Mid Cingulate Cortex
220	R_Dorsal Area 24d	Sensorimotor Associated Paracentral Lobular and Mid Cingulate Cortex
221	R_Ventral Area 24d	Sensorimotor Associated Paracentral Lobular and Mid Cingulate Cortex
222	R_Lateral Area 7A	Superior Parietal and IPS Cortex
223	R_Supplementary and Cingulate Eye Field	Sensorimotor Associated Paracentral Lobular and Mid Cingulate Cortex
224	R_Area 6m anterior	Sensorimotor Associated Paracentral Lobular and Mid Cingulate Cortex
225	R_Medial Area 7A	Superior Parietal and IPS Cortex
226	R_Lateral Area 7P	Superior Parietal and IPS Cortex
227	R_Area 7PC	Superior Parietal and IPS Cortex
228	R_Area Lateral IntraParietal ventral	Superior Parietal and IPS Cortex
229	R_Ventral IntraParietal Complex	Superior Parietal and IPS Cortex
230	R_Medial IntraParietal Area	Superior Parietal and IPS Cortex
231	R_Area 1	Somatosensory and Motor Cortex
232	R_Area 2	Somatosensory and Motor Cortex

233	R_Area 3a	Somatosensory and Motor Cortex
234	R_Dorsal area 6	Premotor Cortex
235	R_Area 6mp	Sensorimotor Associated Paracentral Lobular and Mid Cingulate Cortex
236	R_Ventral Area 6	Premotor Cortex
237	R_Area Posterior 24 prime	Anterior Cingulate and Medial Prefrontal Cortex
238	R_Area 33 prime	Anterior Cingulate and Medial Prefrontal Cortex
239	R_Anterior 24 prime	Anterior Cingulate and Medial Prefrontal Cortex
240	R_Area p32 prime	Anterior Cingulate and Medial Prefrontal Cortex
241	R_Area a24	Anterior Cingulate and Medial Prefrontal Cortex
242	R_Area dorsal 32	Anterior Cingulate and Medial Prefrontal Cortex
243	R_Area 8BM	Anterior Cingulate and Medial Prefrontal Cortex
244	R_Area p32	Anterior Cingulate and Medial Prefrontal Cortex
245	R_Area 10r	Anterior Cingulate and Medial Prefrontal Cortex
246	R_Area 47m	Orbital and Polar Frontal Cortex
247	R_Area 8Av	Dorsolateral Prefrontal Cortex
248	R_Area 8Ad	Dorsolateral Prefrontal Cortex
249	R_Area 9 Middle	Anterior Cingulate and Medial Prefrontal Cortex
250	R_Area 8B Lateral	Dorsolateral Prefrontal Cortex
251	R_Area 9 Posterior	Dorsolateral Prefrontal Cortex
252	R_Area 10d	Orbital and Polar Frontal Cortex
253	R_Area 8C	Dorsolateral Prefrontal Cortex
254	R_Area 44	Inferior Frontal Cortex
255	R_Area 45	Inferior Frontal Cortex
256	R_Area 47l (47 lateral)	Inferior Frontal Cortex

257	R_Area anterior 47r	Orbital and Polar Frontal Cortex
258	R_Rostral Area 6	Premotor Cortex
259	R_Area IFJa	Inferior Frontal Cortex
260	R_Area IFJp	Inferior Frontal Cortex
261	R_Area IFSp	Inferior Frontal Cortex
262	R_Area IFSa	Inferior Frontal Cortex
263	R_Area posterior 9-46v	Dorsolateral Prefrontal Cortex
264	R_Area 46	Dorsolateral Prefrontal Cortex
265	R_Area anterior 9-46v	Dorsolateral Prefrontal Cortex
266	R_Area 9-46d	Dorsolateral Prefrontal Cortex
267	R_Area 9 anterior	Dorsolateral Prefrontal Cortex
268	R_Area 10v	Anterior Cingulate and Medial Prefrontal Cortex
269	R_Area anterior 10p	Orbital and Polar Frontal Cortex
270	R_Polar 10p	Orbital and Polar Frontal Cortex
271	R_Area 11l	Orbital and Polar Frontal Cortex
272	R_Area 13l	Orbital and Polar Frontal Cortex
273	R_Orbital Frontal Complex	Orbital and Polar Frontal Cortex
274	R_Area 47s	Orbital and Polar Frontal Cortex
275	R_Area Lateral IntraParietal dorsal	Superior Parietal and IPS Cortex
276	R_Area 6 anterior	Premotor Cortex
277	R_Inferior 6-8 Transitional Area	Dorsolateral Prefrontal Cortex
278	R_Superior 6-8 Translational Area	Dorsolateral Prefrontal Cortex
279	R_Area 43	Posterior Opercular Cortex
280	R_Area OP4/PV	Posterior Opercular Cortex
281	R_Area OP1/SII	Posterior Opercular Cortex
282	R_Area OP2-3/VS	Posterior Opercular Cortex
283	R_Area 52	Insular and Frontal Opercular Cortex
284	R_RetroInsular Cortex	Early Auditory Cortex
285	R_Area PFcm	Posterior Opercular Cortex

286	R_Posterior Insular Area 2	Insular and Frontal Opercular Cortex
287	R_Area TA2	Auditory Association Cortex
288	R_Frontal OPercular Area 4	Insular and Frontal Opercular Cortex
289	R_Middle Insular Area	Insular and Frontal Opercular Cortex
290	R_Pirform Cortex	Insular and Frontal Opercular Cortex
291	R_Anterior Ventral Insular Area	Insular and Frontal Opercular Cortex
292	R_Anterior Agranular Insula Complex	Insular and Frontal Opercular Cortex
293	R_Frontal OPercular Area 1	Posterior Opercular Cortex
294	R_Frontal OPercular Area 3	Insular and Frontal Opercular Cortex
295	R_Frontal OPercular Area 2	Insular and Frontal Opercular Cortex
296	R_Area PFt	Inferior Parietal Cortex
297	R_Anterior IntraParietal Area	Superior Parietal and IPS Cortex
298	R_Entorhinal Cortex	Medial Temporal Cortex
299	R_PreSubiculum	Medial Temporal Cortex
300	R_Hippocampus	Medial Temporal Cortex
301	R_ProStriate Area	Posterior Cingulate Cortex
302	R_Perirhinal Ectorhinal Cortex	Medial Temporal Cortex
303	R_Area STGa	Auditory Association Cortex
304	R_ParaBelt Complex	Early Auditory Cortex
305	R_Auditory 5 Complex	Auditory Association Cortex
306	R_ParaHippocampal Area 1	Medial Temporal Cortex
307	R_ParaHippocampal Area 3	Medial Temporal Cortex
308	R_Area STSd anterior	Auditory Association Cortex
309	R_Area STSd posterior	Auditory Association Cortex
310	R_Area STSv posterior	Auditory Association Cortex

311	R_Area TG dorsal	Lateral Temporal Cortex
312	R_Area TE1 anterior	Lateral Temporal Cortex
313	R_Area TE1 posterior	Lateral Temporal Cortex
314	R_Area TE2 anterior	Lateral Temporal Cortex
315	R_Area TF	Lateral Temporal Cortex
316	R_Area TE2 posterior	Lateral Temporal Cortex
317	R_Area PHT	Lateral Temporal Cortex
318	R_Area PH	MT+ Complex and Neighbors
319	R_Area TemporoParietoOccipital Junction 1	Temporal-Parietal-Occipital Junction
320	R_Area TemporoParietoOccipital Junction 2	Temporal-Parietal-Occipital Junction
321	R_Area TemporoParietoOccipital Junction 3	Temporal-Parietal-Occipital Junction
322	R_Dorsal Transitional Visual Area	Posterior Cingulate Cortex
323	R_Area PGp	Inferior Parietal Cortex
324	R_Area IntraParietal 2	Inferior Parietal Cortex
325	R_Area IntraParietal 1	Inferior Parietal Cortex
326	R_Area IntraParietal 0	Inferior Parietal Cortex
327	R_Area PF opercular	Inferior Parietal Cortex
328	R_Area PF Complex	Inferior Parietal Cortex
329	R_Area PFm Complex	Inferior Parietal Cortex
330	R_Area PGi	Inferior Parietal Cortex
331	R_Area PGs	Inferior Parietal Cortex
332	R_Area V6A	Dorsal Stream
333	R_VentroMedial Visual Area 1	Ventral Stream
334	R_VentroMedial Visual Area 3	Ventral Stream

335	R_ParaHippocampal Area 2	Medial Temporal Cortex
336	R_Area V4t	MT+ Complex and Neighbors
337	R_Area FST	MT+ Complex and Neighbors
338	R_Area V3CD	MT+ Complex and Neighbors
339	R_Area Lateral Occipital 3	MT+ Complex and Neighbors
340	R_VentroMedial Visual Area 2	Ventral Stream
341	R_Area 31pd	Posterior Cingulate Cortex
342	R_Area 31a	Posterior Cingulate Cortex
343	R_Ventral Visual Complex	Ventral Stream
344	R_Area 25	Anterior Cingulate and Medial Prefrontal Cortex
345	R_Area s32	Anterior Cingulate and Medial Prefrontal Cortex
346	R_posterior OFC Complex	Orbital and Polar Frontal Cortex
347	R_Area Posterior Insular 1	Insular and Frontal Opercular Cortex
348	R_Insular Granular Complex	Insular and Frontal Opercular Cortex
349	R_Area Frontal Opercular 5	Insular and Frontal Opercular Cortex
350	R_Area posterior 10p	Orbital and Polar Frontal Cortex
351	R_Area posterior 47r	Inferior Frontal Cortex
352	R_Area TG Ventral	Lateral Temporal Cortex
353	R_Medial Belt Complex	Early Auditory Cortex
354	R_Lateral Belt Complex	Early Auditory Cortex
355	R_Auditory 4 Complex	Auditory Association Cortex
356	R_Area STSv anterior	Auditory Association Cortex
357	R_Area TE1 Middle	Lateral Temporal Cortex
358	R_Para-Insular Area	Insular and Frontal Opercular Cortex

359	R_Area anterior 32 prime	Anterior Cingulate and Medial Prefrontal Cortex
360	R_Area posterior 24	Anterior Cingulate and Medial Prefrontal Cortex
361	L_Amygdala	Subcortical
362	R_Amygdala	Subcortical
363	L_Hippocampus	Subcortical
364	R_Hippocampus	Subcortical
365	L_Accumbens	Subcortical
366	R_Accumbens	Subcortical
367	L_Caudate	Subcortical
368	R_Caudate	Subcortical
369	L_Pallidum	Subcortical
370	R_Pallidum	Subcortical
371	L_Putamen	Subcortical
372	R_Putamen	Subcortical
373	L_Thalamus	Subcortical
374	R_Thalamus	Subcortical
375	BrainStem	Subcortical
376	L_VentralDiencephalon	Subcortical
377	R_VentralDiencephalon	Subcortical
378	L_Cerebellum	Cerebellar
379	R_Cerebellum	Cerebellar

Supplemental Figure 1: Hierarchical Clustering Dendrogram of Behavioral Phenotypes in TLE (*n*=114)

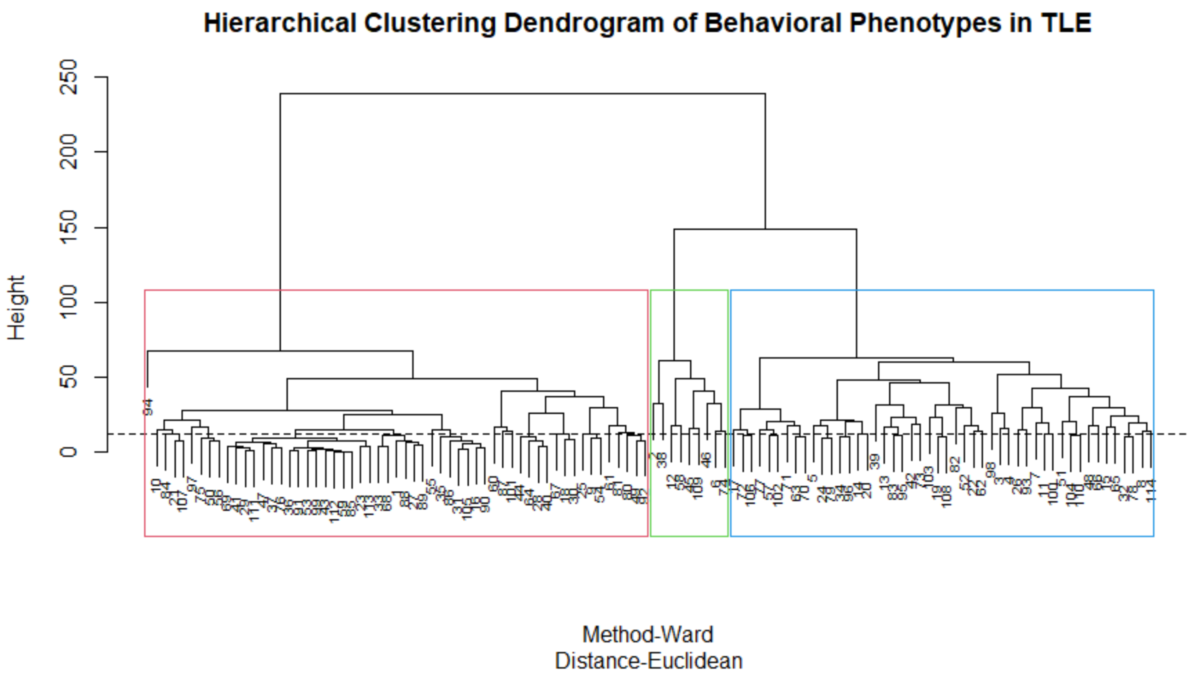

Supplemental Figure 2: Hierarchical clustering: Optimal number of clusters in TLE (n=114) using the Gap statistic method

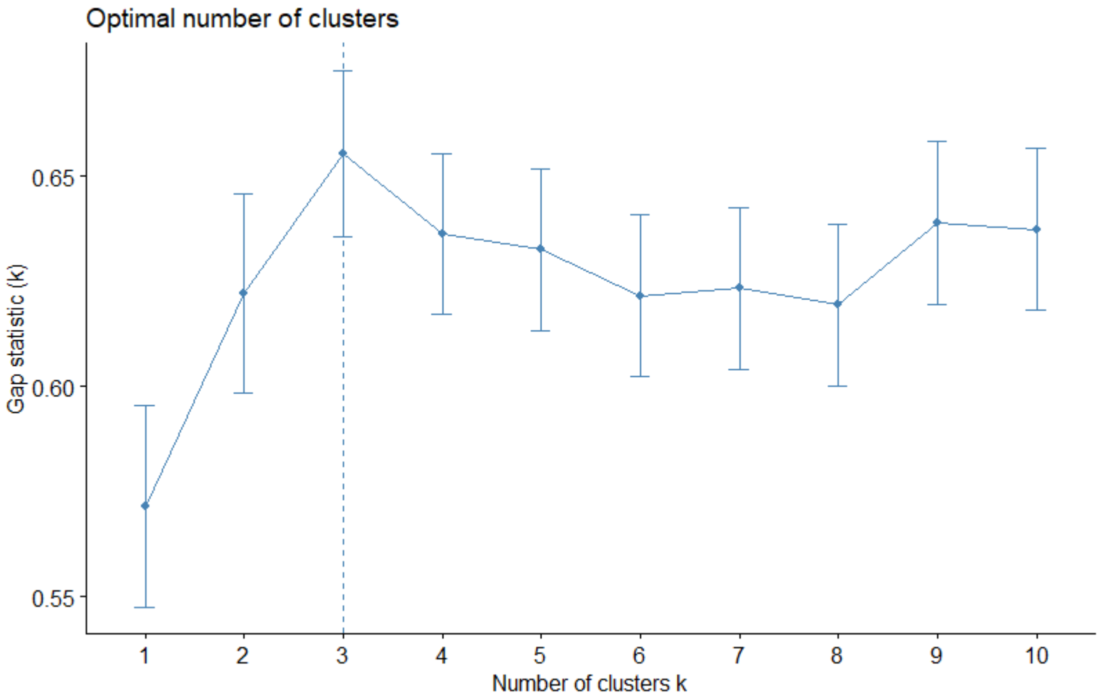

Supplemental Figure 3: Hierarchical Clustering: Principal Component Analysis (PCA) first dimension versus PCA second dimension to visualize separation between groups. The cluster with severe behavioral symptoms in green (triangles) (*n*=24), the cluster with mild behavioral symptoms in red (circles) (*n*=48), and the cluster with no behavioral symptoms in blue (squares) (*n*=42).

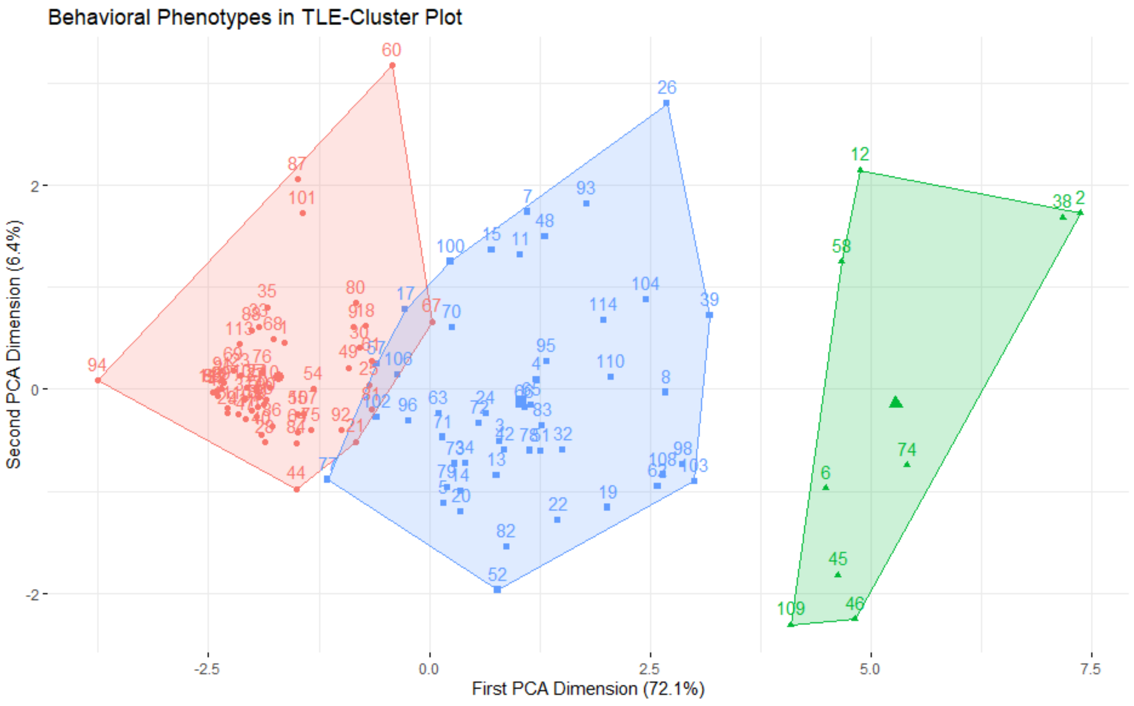

Supplemental Figure 4: Gap Statistic using K-means clustering in TLE (n=114)

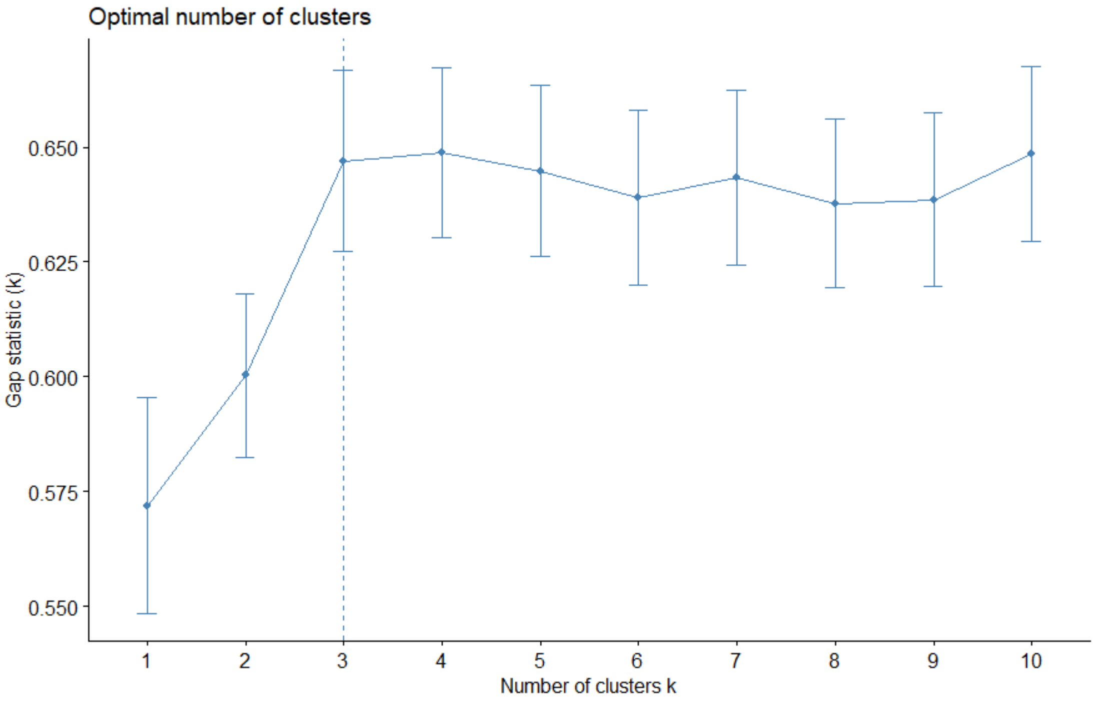

Supplemental Figure 5: Cluster separation using K-means clustering. The cluster with severe behavioral symptoms in yellow (triangles) (*n*=24), the cluster with mild behavioral symptoms in gray (squares) (*n*=48), and the cluster with no behavioral symptoms in blue (circles) (*n*=42)

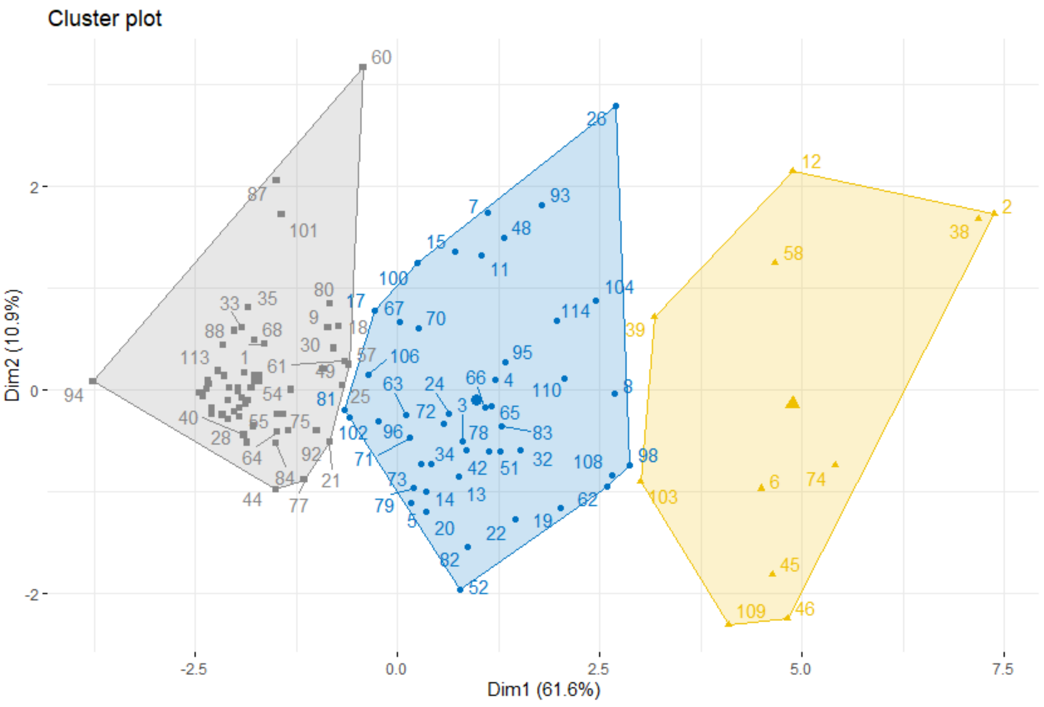

Supplemental Table 3: Overlap Between Hierarchical Clusters and K-Means Clusters

**K-Means Clusters**	**Hierarchical Clusters**			**Total**
	1	2 (mild)	3 (Severe)	
1	55	2	0	57
2 (mild)	2	44	0	46
3 (Severe)	0	2	9	11
**Total**	57	48	9	114

Concordance= 94.7% (6 subjects differ)
